# *LINC00857* knockdown inhibits cell proliferation and induces apoptosis via involving STAT3 and MET oncogenic proteins in esophageal adenocarcinoma

**DOI:** 10.18632/aging.101953

**Published:** 2019-05-13

**Authors:** Wenmei Su, Lihui Wang, Feiyu Niu, Lei Zou, Chunfang Guo, Zhuwen Wang, Xiao Yang, Jiancong Wu, Yi Lu, Jian Zhang, David G. Beer, Zhixiong Yang, Guoan Chen

**Affiliations:** 1 Department of Pulmonary Oncology, Affiliated Hospital of Guangdong Medical University, Zhanjiang 524023, China; 2 Key Laboratory of Longevity and Aging-related Diseases of Chinese Ministry of Education, Center for Translational Medicine & School of Preclinical Medicine, Guangxi Medical University, Nanning, Guangxi 530021, China; 3 Affiliated Cancer Hospital and Institute of Guangzhou Medical University Guangzhou 510000, China; 4 Department of Organ Transplant, First Affiliated Hospital of Kunming Medical University, Kunming 650032, China; 5 Section of Thoracic Surgery, Department of Surgery, University of Michigan, Ann Arbor, Michigan 48109, USA; 6 School of Medicine, Southern University of Science and Technology, Shenzhen 518055, China

**Keywords:** LINC00857, esophageal adenocarcinoma, proliferation, apoptosis

## Abstract

Esophageal adenocarcinoma (EAC) is one of the leading causes of cancer-related death worldwide, and the molecular biology of this cancer remains poorly understood. Recent evidence indicates that long non-coding RNAs are dysregulated in a variety of cancers including EAC. In this study, siRNA mediated gene knockdown, Western blot, RT-PCR, as well as oncogenic function assay were performed. We found that the cell proliferation, colony formation, invasion and migration were decreased after *LINC00857* knockdown in EAC cell lines. We also found that knockdown *LINC00857* could induce apoptosis. Mechanistically, we found that the MET, STAT3, c-Myc and p-CREB proteins were decreased after *LINC00857* knockdown. Our study suggests that *LINC00857* may play an important oncogenic role in EAC via STAT3 and MET signaling.

## INTRODUCTION

Esophageal cancer (EC) is one of the most frequent cancers globally [1], and it carries a poor prognosis with roughly 25% of patients presenting with metastatic disease [2]. There are two main types of EC that occur in the esophagus: esophageal squamous cell carcinoma (ESCC) and esophageal adenocarcinoma (EAC). Rates of esophageal adenocarcinoma have been rising over the past four decades. The reasons for this remarkable increasing are unknown [3, 4]. The mortality rate of EAC is higher worldwide. However, effective strategies to decrease the incidence and mortality of EAC remain lacking.

Accumulating evidence has indicated that long non-coding RNAs (lncRNAs) play important roles in cancer biology [5–9]. Recently, long noncoding RNA (lncRNA) has attracted researchers’ attentions [10]. LncRNAs are involved in various malignant tumors, such as those of the brain [11, 12], breast [13], lung [14, 15], liver [16] and pancreas [17, 18]. LncRNAs, which are defined as being longer than 200 nucleotides without or with limit protein coding ability [19–21], emerge as essential regulators in almost all aspects of biology via regulation at chromatin organization, transcriptional and post-transcriptional levels [22, 23]. Additionally, a number of studies extend our knowledge lncRNAs play important roles in carcinogenesis and cancer metastasis [24–27]. Mounting evidence has shown that lncRNAs are capable of influencing various cellular processes such as cell proliferation, cell cycle regulation, tumor growth and apoptosis [28–31]. Also, despite the vast number of recent lncRNA studies [32, 33], the exact function of lncRNA in esophageal carcinoma tumor genesis is still unknown.

As we know, research on the effects of lncRNA on EAC is still in the preliminary stage, and the related reports are rare [34]. Yang et al [35] reported that dysregulation of *HNF1A-AS1* participated in esophageal tumorigenesis, knockdown of *HNF1A-AS1* inhibited the proliferation and invasion of esophageal adenocarcinoma cells. Wu et al [36] showed that long non-coding RNA *AFAP1-AS1* was reduced in BE and EAC, and its expression inhibited cancer-related biologic functions of EAC cells. Although, there has been a heavy focus on the ways that lncRNAs contribute to cancers development, but their aberrant expression and functional role in EAC development is still not well documented.

*LINC00857* was reported to play oncologic roles in several cancers, for example, lung adenocarcinoma [37], bladder [38], gastric [39, 40] and liver cancer [41], and it has already been suggested that *LINC00857* acts as a cell cycle regulator in lung adenocarcinoma by our previous work [37]. However, the functional role and underlying mechanism of *LINC00857* in EAC remains unclear. Here we investigated the role of *LINC00857* in EAC. We found that knockdown of *LINC00857* decreased cell proliferation, invasion and migration, as well as increased apoptosis in EAC cell lines. The oncogenic role of *LINC00857* may be through multiple oncogenes.

## RESULTS

### *LINC00857* expression was increased in primary EAC and cell lines

**Figure 1:**
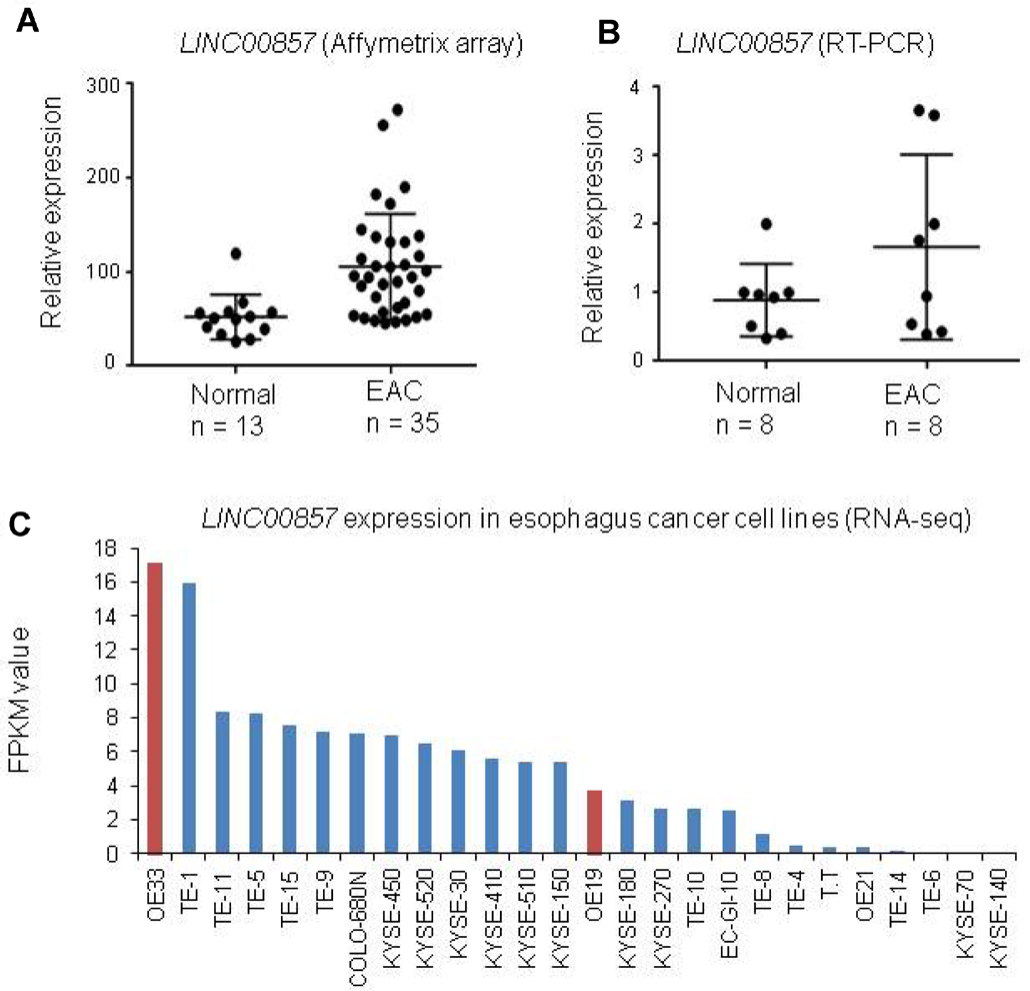
***LINC00857* expression was increased in primary EAC and cell lines.** (**A**) *LINC00857* was increased in EAC as compared to normal esophagus tissues (The original data is coming from our unpublished Affymetrix Human Gene ST2.1 including 35 EAC and 13 paired non-tumor esophageal tissues). (**B**) *LINC00857* expression was higher in EAC by RT-PCR using another set of tissues including 8 EAC and 8 normal esophagus controls. (**C**) *LINC00857* expression in a larger RNA-Seq data [42] including 26 esophagus cancer cell lines.

*LINC00857* expression was increased in many types of cancer [37], but there is no report of this lncRNA expression status in esophagus cancer. By analyzed our esophagus cancer Affymetrix array data (unpublished data), we found *LINC00857* was increased in EAC as compared to normal esophagus tissues (Figure 1A). Next, we performed RT-PCR for *LINC00857* expression using another set of tissues including 8 EAC and 8 normal esophagus controls. We confirmed that *LINC00857* expression was higher in EAC (Figure 1B). We also evaluated the *LINC00857* expression in a larger RNA-Seq data [42] including 26 esophagus cancer cell lines. We found 19/26 cell lines have higher *LINC00857* expression level more than 1 FPKM value, and OE33, an adenocarcinoma, was the highest one (Figure 1C).

### The cell proliferation and colony formation were decreased after knockdown of *LINC00857* in EAC

**Figure 2:**
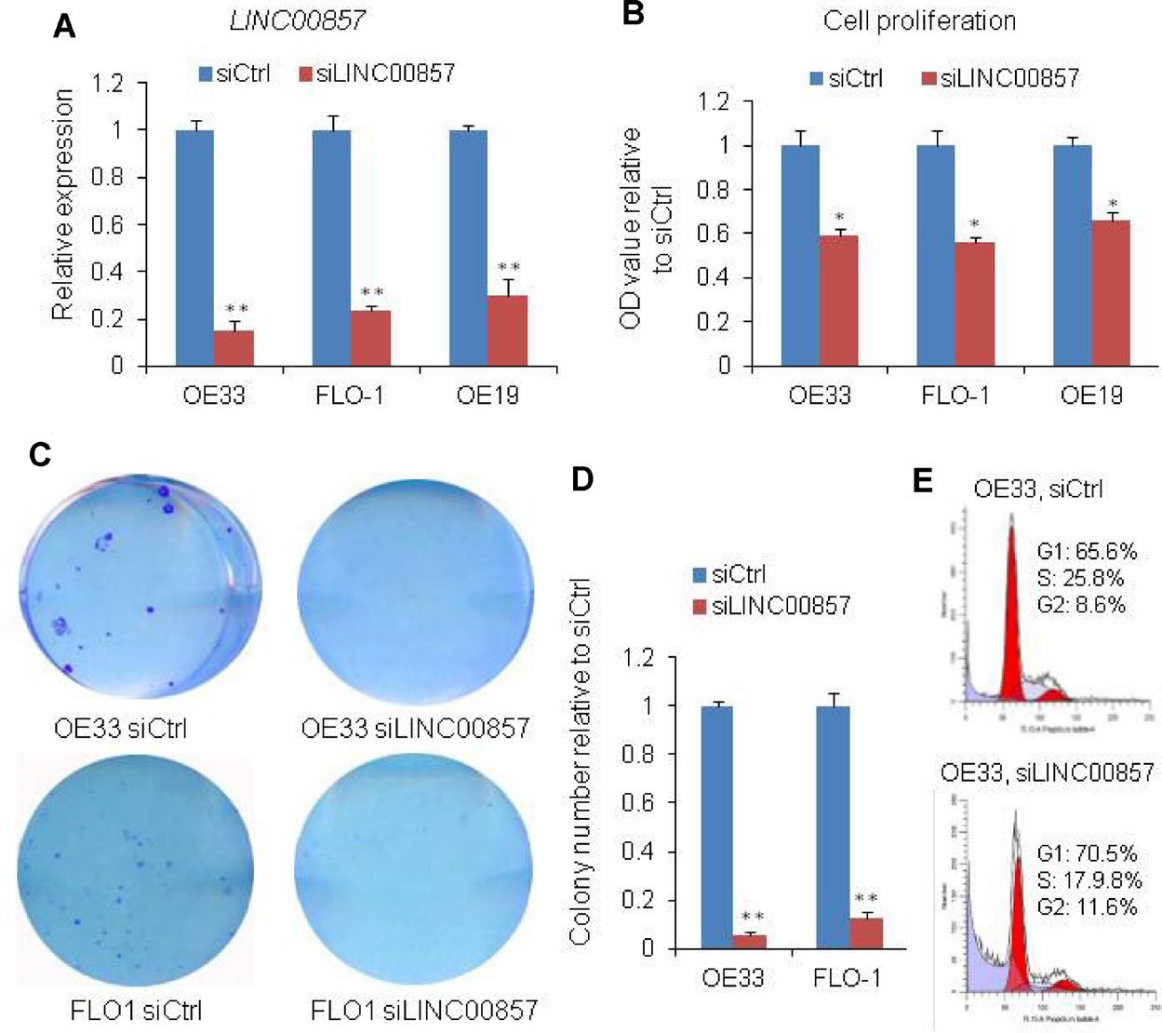
**SiRNA-mediated knockdown of *LINC00857* inhibits EAC cell proliferation.** (**A**) The *LINC00857* expression level was determined by qPCR when OE19, OE33 and FLO1 cells transfected with *siLINC00857*. (**B**) WST-1 assays were used to determine the cell viability for *siLINC00857*-transfected OE33, FLO1 and OE19. (**C**) Colony-forming assays were conducted to determine the colony formation of *siLINC00857*-transfected OE33 and FLO1 cells. (**D**) The bar chart represented the count number of cloning. Values represented the mean ± s.d. from three independent experiments. *p < 0.05, **p < 0.01. (**E**) Flow cytometry analysis showed that the cell cycle was arrested at G1 phase after *LINC00857* knockdown in OE33 cells.

*LINC00857* was reported to play an oncogenic role in lung cancer. To test if *LINC00857* was functionally involved in EAC, we measured cell proliferation and colony formation followed by *LINC00857* knockdown with siRNAs in 3 EAC cell lines, OE19, OE33 and FLO1. QRT-PCR assays revealed that *LINC00857* expression was significantly reduced after transfection with *LINC00857* siRNAs (**p < 0.01, Figure 2A). The cell proliferation was performed using WST-1 assay and the result showed that the cell proliferation was decreased by more than 35% after *LINC00857* knockdown at 120 h in OE19, OE33 and FLO1 cell lines (*p < 0.05, Figure 2B). Similarly, the colony-formation was significantly decreased following inhibition of *LINC00857* in OE33 and FLO1 cell lines (**p < 0.01, Figures 2C and 1D). Flow cytometry analysis indicated that the cell cycle was arrested at G1 phase after *LINC00857* knockdown in OE33 cells (Figure 2E). These findings suggested that *LINC00857* was involved in the regulation of cell proliferation/cell cycle in EAC cells.

### Cell migration and invasion were decreased after *LINC00857* knockdown in EAC cells

**Figure 3:**
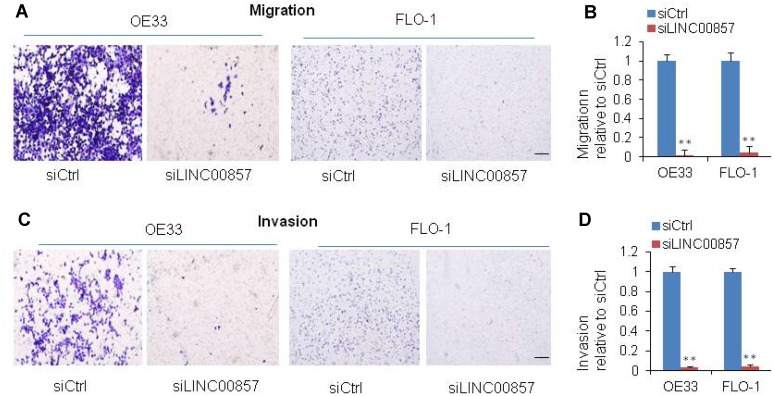
**SiRNA-mediated knockdown of *LINC00857* inhibits EAC cell migration and invasion.** (**A**) Migration was decreased after *LINC00857* siRNA transfection of OE33 and FLO1 cells. (**B**) The bar chart represented the count number of migration cells. (**C**) Invasion was decreased after *LINC00857* siRNA transfection of OE33 and FLO1 cells. (**D**) The bar chart represented the count number of invasion cells. Values represented the mean ± s.d. from three independent experiments. *p < 0.05, **p < 0.01.

Cell migration and invasion are significant aspects of cancer progression, which involves the dissolution of extracellular matrix proteins and the migration of tumor cells into contiguous tissues. In order to investigate the potential role of *LINC00857* on cell migration and invasion in EAC cells, we performed transwell assays. We found that the cell migration was inhibited by 90% after *LINC00857* knockdown in OE33 and FLO-1 cells (**p < 0.01, Figure 3A and 3B). The cell invasion was down-regulated by 90% in OE33 and FLO-1 followed *LINC00857* knockdown (**p < 0.01, Figure 3C and 3D). These results implied that *LINC00857* may be involved in mechanisms relevant to the metastatic potential of EAC.

### Apoptosis was induced after *LINC00857* knockdown in EAC cells

**Figure 4:**
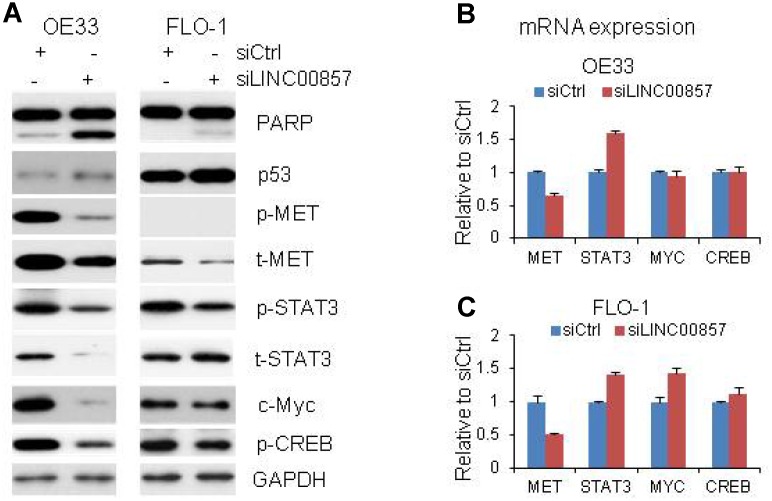
**Proteins and mRNA regulated by *LINC00857*.** (**A**) Protein levels of p53, Cleavage of PARP, MET, STAT3, c-Myc and CREB were regulated by *LINC00857* siRNA in OE33 and FLO cells, GAPDH was used as a protein loading control. (**B** and **C**) QRT-PCR showing the mRNA expression of MET, STAT3, c-Myc and CREB in OE33 and FLO1 cells. GAPDH was used as control.

To probe potential role of *LINC00857* in cell death regulation, we assessed apoptosis assay by measuring cleavedPARP protein in OE33 and FLO1 cells. Western blot indicated that the cleavedPARP bands (Figure 4A) occurred only after *LINC00857* siRNA treatment, suggesting that inhibition of *LINC00857* could induce apoptosis in these cell lines. Moreover, *LINC00857* knockdown led to an increased expression of p53, suggesting this apoptosis may be p53 mediated.

### Multiple oncogenic proteins including MET, STAT3, c-Myc and CREB were decreased upon *LINC00857* knockdown

In a further attempt to dissect potential molecular signaling regulated by *LINC00857,* we applied Western blot to identify proteins whose expression altered after *LINC00857* knockdown in OE33 and FLO1 cells. We found that several oncogenic proteins including t-MET, p-MET, p-STAT3, c-Myc and p-CREB were decreased after *LINC00857* knockdown with siRNA treated cells at 72 hrs in OE33 and FLO1 cells (Figure 4A). The t-STAT3 was not decrease in FLO1 cells. The results indicated that these proteins may take an important role in regulating cell proliferation and apoptosis in EAC cells in *LINC00857* network. We performed the mRNA expression of these genes by RT-PCR, we found the MET mRNA was down regulated by 40–50% in both OE33 and FLO1 cells, indicating the MET gene was regulated at the transcriptional level upon *LINC00857* knockdown (Figure 4B and 4C). While, the mRNA levels of STAT3, c-Myc and CREB were either increased or not changed after *LINC00857* knockdown, suggesting these proteins were regulated at post-transcriptional level. We didn’t find that ERK, AKT, FAK, p27, and Bax proteins were changed after *LINC00857* siRNA treatment at 72 hours in EAC.

**Figure 5:**
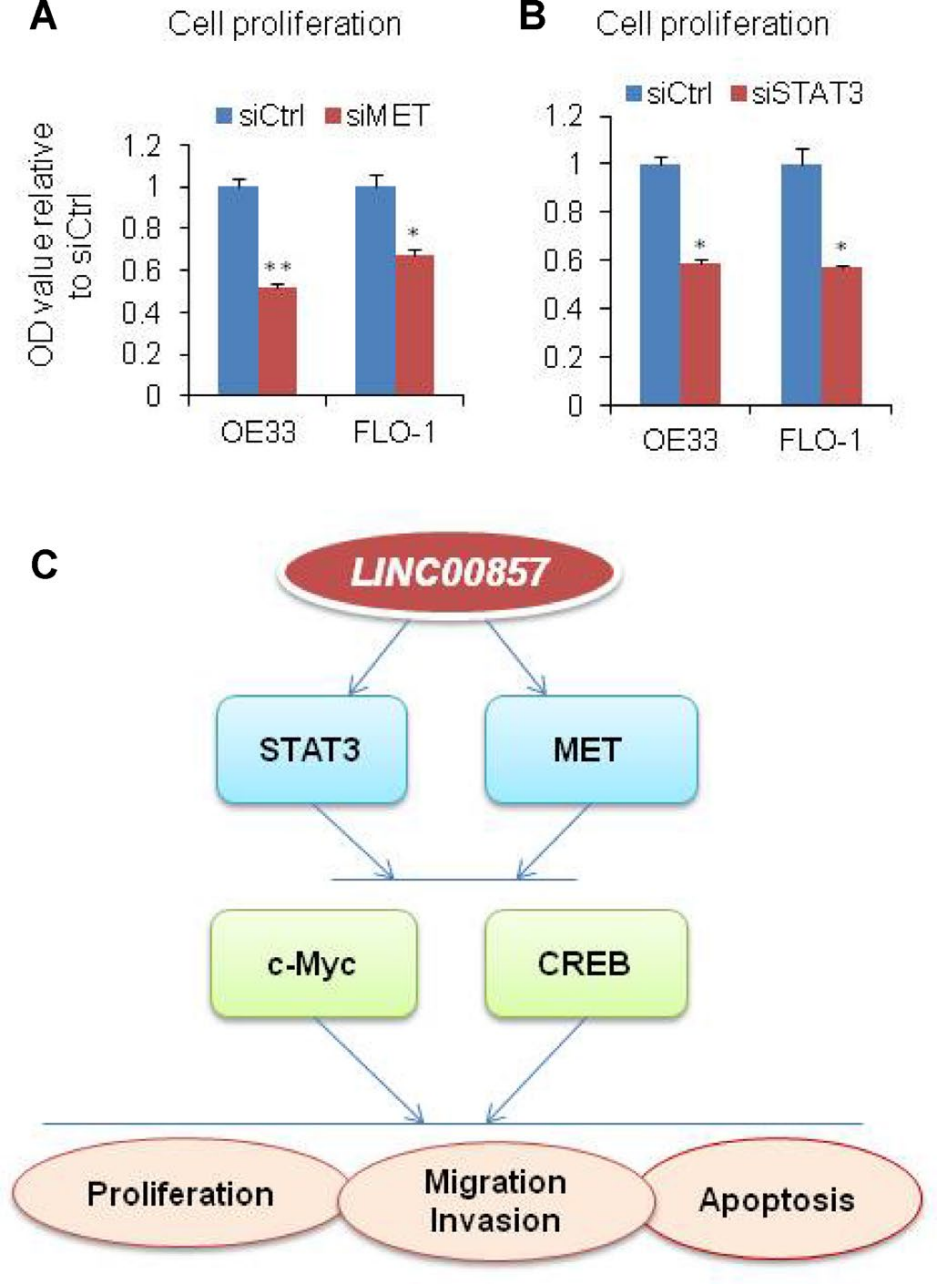
**Model of *LINC00857* in EAC.** (**A** and **B**) WST-1 assays were used to determine the cell viability for MET and STAT3 siRNA transfecting OE33 and FLO1 cells. Values represented the mean ± s.d. from three independent experiments. *p < 0.05, **p < 0.01. (**C**) Model of *LINC00857* modulating the cells proliferation, migration, invasion and induced apoptosis via MET, STAT3, c-Myc and CREB proteins in EAC.

OE33 is known as MET driven cell, in order to evaluate the role of MET in EAC cells, we performed MET siRNA knockdown on OE33 and FLO1cells. After MET knockdown, the cell proliferation measured by WST-1 assay was decreased by more than 33%–49% relative to control scramble siRNA (Figure 5A). We also found that the cell proliferation was decreased upon STAT3 knockdown (Figure 5B). This suggested that MET and STAT3 are important oncogenes in *LINC00857* regulating cancer progression in EAC cells. Taken together, *LINC00857* affecting tumor cell proliferation, colony formation, apoptosis, as well as migration and invasion may be via MET, STAT3, c-Myc and CREB oncoproteins (Figure 5C).

## DISCUSSION

*LINC00857* is a novel lncRNA transcribed from chromosome 11q22.3, and played an oncogenic role in lung cancer [37]. *LINC00857* was also reported to be detectable in patient plasma with gastric cancer [40]. The oncogenic role of this lncRNA in EAC has not been reported. Our study showing that the *LINC00857* was overexpressed in EAC tissues and EAC cell lines. We found that the cell proliferation and colony formation were decreased after *LINC00857* knockdown with siRNA in EAC cells. The cell apoptosis was also induced upon *LINC00857* knockdown, indicating *LINC00857* may be involved in EAC tumor growth. Metastasis is another important malignant behavior of cancer and is the most troublesome problem in tumor prognosis and therapy. LncRNAs have been reported to involve in the regulation of tumor metastasis such as MALAT-1 [43] and HOX antisense intergenic RNA (HOTAIR) [44]. We found that the cell migration and invasion ability of EAC cells were significantly decreased after the knockdown of *LINC00857*, suggesting *LINC00857* may be involved in the regulator of metastasis in EAC.

*LINC00857* induces tumorigenesis through complicated mechanisms, involving activation of signaling pathways that regulate cell survival and proliferation. Previous studies have shown that downregulation of *LINC00857* was able to restrain HCC cell migration and invasion capacity via enhancing epithelial-mesenchymal transition (EMT) process [41]. *LINC00857* knockdown decreased protein expression of cyclin D1 and cyclin E1 in GC cells [39]. Wang [37] shows that *LINC00857* mediated tumor progression via cell cycle regulation in lung cancer. In this study, mechanistically, we found that multiple oncogenic proteins such as MET, STAT3, c-Myc and CREB were decreased upon *LINC00857* knockdown in EAC cells. Knockdown MET or STAT3, the cell proliferation were also decreased indicating these two proteins may be involved in *LINC00857* signaling in regulation of EAC cell proliferation. According to recently reports, Stat3 /c-Myc signaling pathways induced tumor development in gastric cancer [45]. c-Met strongly increased the level of transgenic c-Myc that was expressed via a constitutive CMV promoter in large-cell medulloblastoma [46]. These results are supporting evidence that *LINC00857* abrogation induced apoptosis and decreased migration and invasion ability maybe through the inhibition of MET or STAT3 signaling.

In summary, *LINC00857* influenced tumor cell proliferation, colony formation, apoptosis, as well as migration and invasion which may be via affecting MET/STAT3/c-Myc/CREB oncoproteins (Figure 5C). These findings suggested that *LINC00857* is a functional lncRNA in human EAC cells and plays an important role in EAC progression.

## MATERIALS AND METHODS

### Cell culture

EAC cell lines OE19, OE33 and FLO1 were purchased from Sigma Chemical (St Louis, Missouri, USA), and the European Collection of Cell Culture (Porton Down, UK), respectively. All media were supplemented with 10% fetal bovine serum (Invitrogen, San Diego, California, USA), unless otherwise stated.

### Esophageal cancer specimen

Esophageal adenocarcinoma tissues and paired non-tumor tissues were obtained from patients undergoing cancer surgery during the period from 1995 to 2013 at the University of Michigan Health System. None of the patients included in this study received any preoperative radiation or chemotherapy. All the patients provided informed consent, and all experimental protocols were approved by the University of Michigan Institutional Review Board and Ethics Committee. Resected specimens were frozen in liquid nitrogen and then stored at -80°C until use. Frozen tissues for regions containing a minimum of 70% tumor cellularity defined by cryostat sectioning were utilized for RNA isolation.

### Cell Proliferation Assays

Cells were plated at a density of 1000 cells per well onto 96-well plates. *LINC00857* siRNA and control siRNA were added at 24 hrs and 48 hrs. Cell proliferation was measured at 96 hrs-120 hrs after siRNA transfection using WST-1 reagent (Roche, Mannheim, Germany) according to manufacturer’s instructions. All experiments were performed in triplicate.

### RNA isolation and qRT-PCR

Total RNA was isolated from tissues or cultured cells using miRNeasy Mini kit (Qiagen) according to the manufacturers' instructions. One microgram of total RNA was used for the reverse transcription reaction in a final volume of 20 *μ*L with random primers under standard conditions using High Capacity cDNA Reverse Transcription kit (Thermo Fisher Scientific). 1 *μ*L of the corresponding cDNA was used for subsequent qRT-PCR reactions using Power SYBR Green master Mix (Thermo Fisher Scientific) according to the manufacturer’s instructions. The expression of GAPDH was used to normalize the results. The PCR amplification was performed for 40 cycles of 95°C for 15 sec and 60°C for 60 sec on StepOne Real-Time PCR System (Thermo Fisher Scientific). All reactions were run in triplicate and data were analyzed using the comparative cycle threshold (CT) method. The primer sequences are summarized in Table 1.

**Table 1:** Primer sequences used in this study.

**Gene name**	**Primer ID**	**Primer Sequence (5'->3')**	**Product size**
LINC00857	Lnc1 F	CCCCTGCTTCATTGTTTCCC	131
LINC00857	Lnc1 R	AGCTTGTCCTTCTTGGGTACT	
MYC	MYC F	CAGCGACTCTGAGGAGGAAC	131
MYC	MYC R	TGTGAGGAGGTTTGCTGTGG	
STAT3	STAT3_F	TGGCCCAATGGAATCAGCTAC	200
STAT3	STAT3_R	CTGCTGGTCAATCTCTCCCA	
MET	MET F	CAACCCGAATACTGCCCAGA	99
MET	MET R	CCGGGACACCAGTTCAGAAA	
CREB	CREB F	GCACTATTGCCCCTGGAGTT	127
CREB	CREB R	CTACGACACTCTCGAGCTGC	
GAPDH	GAPDH gcF	GTCAAGGCTGAGAACGGGAA	158
GAPDH	GAPDH gcR	AAATGAGCCCCAGCCTTCTC	

### Cell migration and invasion assay

Migration and invasion capabilities of the esophageal cells were measured in transwell chamber system. Sixty microliters of diluted extracellular matrix (ECM) gel solution was added into the upper chambers (Costar Inc., USA). For migration assay, the same method was used without ECM membrane on the upper chamber. The chamber was incubated at 37 °C for 4 h. Next, a density of 1×105 per well cells was seeded in 100-μl medium with 1% FBS. The lower level chambers of the transwells were filled with 500 μl RPMI and DMEM containing 10% FBS. To allow cell migration, the transwells were then incubated at 37 °C with 5 % CO2 for 24-48 h. After incubation, a cotton swab was used to carefully remove the cells in the upper chamber. Cells at the bottom of the ECM gel-coated membrane were fixed and stained with paraformaldehyde and crystal violet. The crystal violet was dissolved in 200 μl glacial acetic acid and incubated at room temperature for 30 min.

### Colony formation assay

Esophageal cells were cultured with siRNA mock and *LINC00857* siRNA and then seeded in a six-well plate at a density of 200 cells / well. After a 2-week period of incubation at 37 °C, 0.1 % crystal violet (Sigma-Aldrich) and 20 % methanol were used as dye solution to fix and stain the colonies. The number of colonies was counted in each well. Clones containing more than 50 cells were counted using a grid. Three independent experiments were performed. The formula for the colony formation ratio was as follows: Ratio = Numbers of Colony/Initiative Cells × 100%.

### Western blotting

Cells were harvested 72 h after siRNA transfection. Lysis, electrophoresis and target protein visualisation were performed as described previously [37]. Total cell lysates were prepared with sample buffer and boiled at 95 °C for 5 min. The samples were transferred to SDS–PAGE at 80 V for 3 h and then transferred to PVDF membranes for another 3 h. After incubation with specific antibodies for STAT3, FAK, PARP, c-Myc, CREB, MET, AKT, ERK1/2 and GAPDH at 4 °C overnight, the membranes then were washed by 1% TBST for three times, incubated with secondary antibodies for 1 h, and the membranes were developed using ECL and exposed to X-ray film.

### Statistical analysis

Data were analyzed using GraphPad Prism 7 (GraphPad software) and R software. All data are continuous variables and follow a normal distribution. The other data such as proliferation were evaluated by unpaired Student’s t-test. All values were expressed as mean±SD. Statistical significance was noted at p<0.05. Three independent triplicated experiments were performed for cell biological assays, unless otherwise stated.
